# Case Report: From suspected congenital rubella to a lysosomal storage disorder—diagnostic challenges in a neonate with findings strongly suggestive of Gaucher disease

**DOI:** 10.3389/fped.2026.1877098

**Published:** 2026-09-25

**Authors:** Ruifeng Tian, Shiwen Xia

**Affiliations:** Department of Neonatology, Maternal and Child Health Hospital of Hubei Province, Tongji Medical College, Huazhong University of Science and Technology, Wuhan, Hubei, China

**Keywords:** case report, congenital rubella, diagnostic uncertainty, Gaucher disease, lysosomal storage disorder, neonate

## Abstract

**Background:**

Neonatal lysosomal storage disorders may overlap clinically with congenital infection. We report a term infant initially evaluated for congenital rubella because of petechiae, thrombocytopenia, hepatosplenomegaly, cholestasis, bilateral non-pass results on newborn hearing screening, cardiac abnormalities, and ocular findings.

**Case presentation:**

A term male neonate presented with petechiae, gastrointestinal bleeding, thrombocytopenia, hepatosplenomegaly, cholestasis, liver dysfunction, bilateral non-pass newborn hearing-screening results, cardiac abnormalities, and ocular findings after repeated maternal rubella immunoglobulin M and G positivity during pregnancy. Infant rubella immunoglobulin G was positive and immunoglobulin M was negative, but rubella-specific reverse-transcription polymerase chain reaction and adequate longitudinal serology were not performed. Dried-blood-spot (DBS) glucocerebrosidase activity was 0.96 μmol/L/h (reference interval, 1.19–22.23), only modestly reduced, whereas glucosylsphingosine (lyso-Gb1) was 963.66 ng/mL (reference value, <17.41), more than 55-fold the upper limit. Neither result was confirmed in an independently collected specimen, and no leukocyte or fibroblast enzyme assay was performed. Long-range polymerase chain reaction and sequencing reported *GBA1* c.1265_1319del as apparently homozygous, but parental Sanger testing detected the variant in the father and not the mother. Biallelic *GBA1* involvement therefore remained unresolved. Alternative neonatal lysosomal disorders were not systematically excluded. The findings were consistent with probable neonatal-onset Gaucher disease but did not establish a definitive diagnosis. The infant was discharged after 21 days and was reported to have died at 6 months; the intervening course and certified cause of death were unavailable.

**Conclusion:**

This case illustrates diagnostic uncertainty in neonates with overlapping infectious, haematological, inflammatory, and lysosomal features. Markedly elevated lyso-Gb1 provided the strongest Gaucher-directed biochemical evidence, whereas the modestly reduced single dried-blood-spot enzyme result and unresolved GBA1 segregation precluded definitive confirmation. Relevant Gaucher-like and hepatosplenic lysosomal disorders were not systematically excluded. The findings were consistent with probable neonatal-onset Gaucher disease; congenital rubella remained unconfirmed and could not be fully excluded, and the disease subtype, allelic configuration, inheritance mechanism, and recurrence risk remained undetermined.

## 1 Introduction

Gaucher disease is an autosomal recessive lysosomal storage disorder caused by deficient glucocerebrosidase activity, most often due to biallelic pathogenic variants in *GBA1* (1, 2). Severe neonatal presentations may include hepatosplenomegaly, cytopenias, cholestasis, respiratory compromise, skin abnormalities, and neurological involvement (1–3).

Congenital rubella can produce purpura, hepatosplenomegaly, thrombocytopenia, congenital heart disease, hearing impairment, and ocular abnormalities (4). These features also overlap with haemophagocytic lymphohistiocytosis (HLH), disseminated intravascular coagulation (DIC), immune-mediated thrombocytopenia, and inherited metabolic disease.

We report a neonate initially evaluated for congenital rubella whose phenotype and laboratory findings were strongly suggestive of neonatal-onset GD, although independent biochemical confirmation and unequivocal demonstration of biallelic GBA1 involvement were lacking. The principal educational message is the need to preserve diagnostic uncertainty while evaluating rare multisystem neonatal disorders with overlapping infectious, haematological, inflammatory, and metabolic features.

## 2 Case description

### 2.1 Patient information and initial findings

The 31-year-old mother conceived by *in vitro* fertilisation. Rubella immunoglobulin M and G were reported as positive at 11 weeks and 4 days of gestation (2.76 and 3.68 AU/mL, respectively), 13 weeks and 4 days (4.71 and 3.70 AU/mL), and 16 weeks and 4 days (4.44 and 3.89 AU/mL). She reported no fever, rash, compatible illness, or known rubella exposure; earlier vaccination and immunity records were unavailable. Rubella immunoglobulin G avidity, seroconversion assessment, serial quantitative titre interpretation, and confirmatory maternal immunoglobulin M testing by an independent method were not performed. According to the parents, there was no known family history of lysosomal storage disorders or other inherited metabolic diseases, and no unexplained neonatal or infant deaths, including sudden deaths in infancy, were reported among maternal or paternal relatives.

A male infant was delivered by caesarean section at 39 weeks and 6 days. Birth weight was 3.35 kg, length 50 cm, head circumference 34 cm, and Apgar scores were 9 and 10. Generalised petechiae developed within the first 13 h, most densely over both lower limbs, and gastric decompression yielded coffee-ground/bloody material. Before transfer, platelet count was 45 × 10^9^/L and fibrinogen 0.67 g/L; ceftazidime, etamsylate, vitamin K1, and 50 mL of plasma had been administered. On admission he was alert and reactive, with mild respiratory distress and the liver and spleen palpable approximately 3 cm below the costal margins. Sucking and swallowing were intact, primitive reflexes and limb tone were normal, and no clinically suspected intracranial bleeding was documented. No hydrops, oedema, collodion membrane, ichthyosis, joint contractures, bulbar dysfunction, seizures, abnormal tone, or congenital anomaly was identified during the admission.

Serial testing documented persistent thrombocytopenia, progressive conjugated hyperbilirubinaemia, liver injury, anaemia, neutropenia, fluctuating coagulopathy, and hyperferritinaemia. Platelet count increased from 25 × 10^9^/L on hospital day 1 to 88 × 10^9^/L on day 21, whereas haemoglobin reached a nadir of 104 g/L on days 17 and 19 and was 111 g/L at discharge. Direct bilirubin reached 156.8 μmol/L, total bile acids 152.6 μmol/L, alanine aminotransferase 114 U/L, aspartate aminotransferase 413 U/L, ferritin >2,000 ng/mL, and D-dimer 14.00 mg/L. The lowest fibrinogen concentration was 0.67 g/L before transfer and 1.15 g/L during admission. Serial abdominal ultrasonography and computed tomography confirmed hepatosplenomegaly without a focal mass, portal hypertension, or adrenal calcification; gallbladder contraction was normal on day 14. Echocardiography showed a muscular ventricular septal defect, patent ductus arteriosus, right-heart enlargement, mild tricuspid regurgitation, and pulmonary hypertension with preserved left ventricular systolic function. Cranial ultrasonography and brain magnetic resonance imaging were unremarkable. Automated auditory brainstem-response screening yielded bilateral non-pass results, but diagnostic auditory testing was not performed. Right iris dysplasia and an immature retina were recorded without retinal haemorrhage, exudation, or other findings diagnostic of rubella retinopathy.

### 2.2 Diagnostic assessment

Infant rubella immunoglobulin G was positive and immunoglobulin M negative on hospital days 1 and 13. Blood metagenomic sequencing detected no pathogen but was not a validated rubella-specific test. Rubella-specific reverse-transcription polymerase chain reaction of respiratory or urine specimens and adequate longitudinal infant immunoglobulin G testing were not performed; congenital rubella therefore remained unconfirmed and could not be fully excluded. Blood culture, cerebrospinal-fluid testing, cytomegalovirus nucleic acid testing, and other pathogen-directed investigations were negative. Amino-acid, acylcarnitine, urinary organic-acid, and ammonia screening was unremarkable, but these tests do not exclude lysosomal storage disorders. No disease-directed enzyme, biomarker, or molecular testing for alternative lysosomal storage disorders was performed.

Bone marrow showed active haematopoiesis with decreased megakaryocytes and platelets, without haemophagocytosis, blasts, foam cells, or definite Gaucher cells; the absence of Gaucher cells does not exclude Gaucher disease. Four HLH-2004 criteria were documented at different time points—splenomegaly, cytopenias in two lineages, hyperferritinaemia, and hypofibrinogenaemia—but fever ≥38.5 °C, hypertriglyceridaemia, haemophagocytosis, and haemoglobin below the neonatal threshold were absent. Natural-killer-cell activity, soluble CD25, and familial-HLH testing were not assessed. Because the findings were recorded at different time points and after several interventions, they did not represent a single untreated diagnostic snapshot. HLH was considered but not diagnosed (5). The admission findings met retrospective neonatal DIC criteria, although plasma had already been given before the day-1 assessment (6).

On hospital day 14, dried-blood-spot glucocerebrosidase activity was 0.96 μmol/L/h (reference interval, 1.19–22.23) and lyso-Gb1 was 963.66 ng/mL (reference value, <17.41). Both reports recorded acceptable sample quality. The enzyme result was only modestly below the lower limit, whereas lyso-Gb1 exceeded the upper limit by more than 55-fold. Neither result was repeated in an independently collected specimen, and glucocerebrosidase activity was not measured in leukocytes or fibroblasts. Plasma and platelet transfusions, but no erythrocyte transfusion, preceded sampling. Consequently, the enzyme measurement, biomarker result, and molecular evidence were interpreted as separate components of the diagnostic assessment rather than as interchangeable proof of a confirmed disorder.

Long-range polymerase chain reaction followed by sequencing of the same-day blood-spot sample reported GBA1 NM_001005741.3:c.1265_1319del (also represented on the MANE Select transcript as NM_000157.4:c.1265_1319del; p.Leu422fs) in an apparently homozygous state and classified it as pathogenic (7). Sanger testing of parental dried blood spots detected the variant heterozygously in the father and not in the mother (Figure 1). Clinical records and parental confirmation indicated that the tested adults were the infant's biological parents and that their own gametes had been used for the *in vitro* fertilisation; independent kinship analysis was not performed. The proband's trace-level or read-level data were unavailable. Neither whole-exome sequencing (WES) nor whole-genome sequencing (WGS) was performed in the proband or his parents. No independent copy-number assay, single-nucleotide-polymorphism array, informative-marker analysis, trio sequencing, or long-read analysis was performed. Biallelic GBA1 involvement, allelic configuration, inheritance mechanism, and recurrence risk therefore remained unresolved.

**Figure 1:**
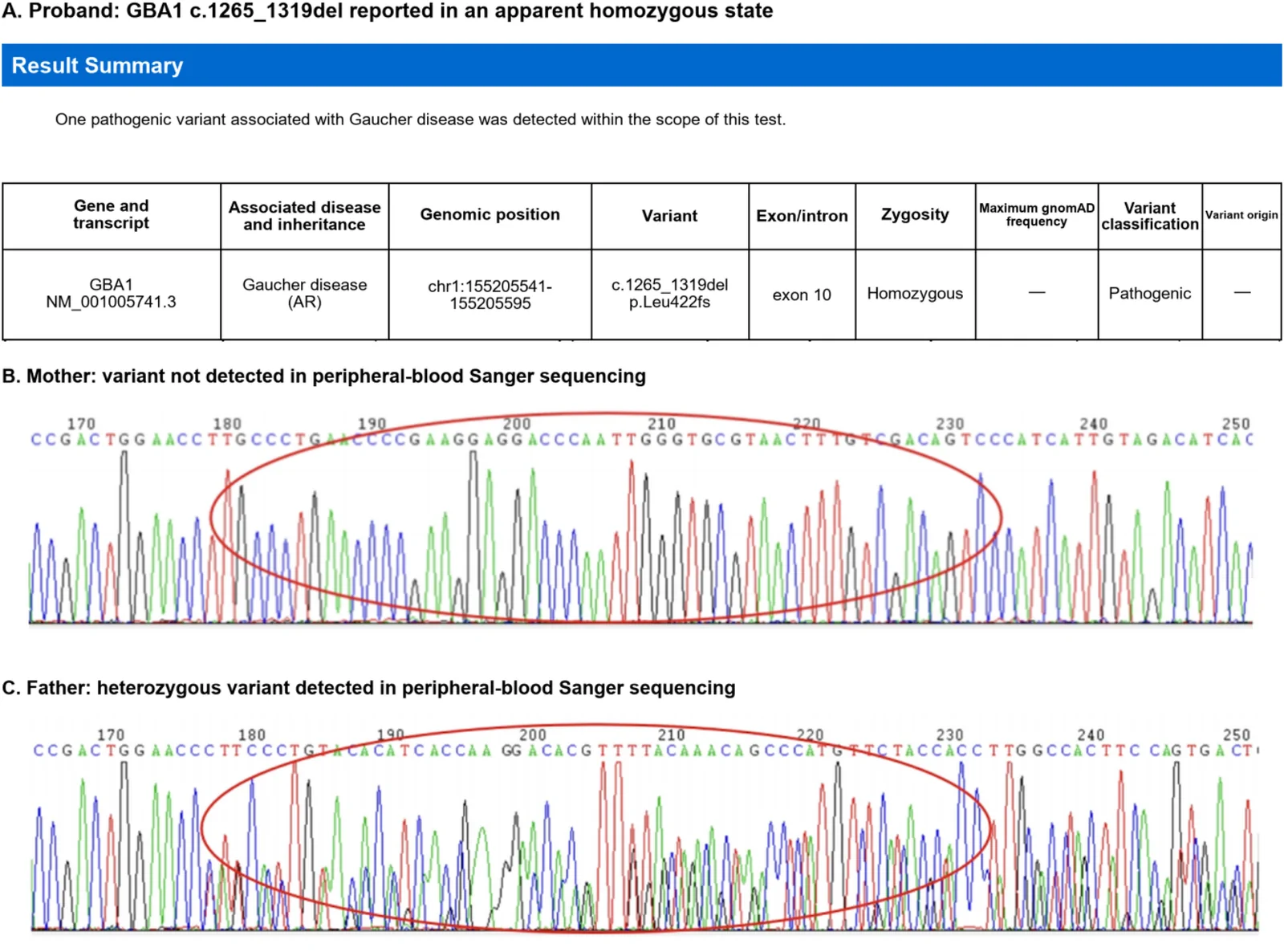
**(A)** The proband's laboratory report identified *GBA1* c.1265_1319del as apparently homozygous and classified it as pathogenic. **(B)** Maternal Sanger sequencing did not detect the variant. **(C)** Paternal Sanger sequencing detected the variant heterozygously. Panel A reproduces the report summary; underlying proband trace-level or read-level data were unavailable. The discordant segregation did not demonstrate biallelic *GBA1* involvement or establish allelic configuration, inheritance mechanism, or recurrence risk.

### 2.3 Therapeutic intervention

Continuous positive airway pressure was provided for 2 days, after which spontaneous breathing remained stable. Empirical meropenem 65 mg every 12 h and ampicillin 160 mg every 8 h were administered for 6 days because severe thrombocytopenia, gastrointestinal bleeding, respiratory distress, and possible neonatal infection were present. Antibiotics were stopped after clinical improvement, a decrease in C-reactive protein from 25.1 mg/L on day 2 to 3.18 mg/L on day 6, and negative microbiological investigations. Eighty millilitres of platelets was transfused for a platelet count of 25 × 10^9^/L with gastrointestinal and cutaneous bleeding; the count increased to 61 × 10^9^/L the following day and the bleeding manifestations improved. Retrospective application of neonatal DIC criteria yielded a score of 5 before plasma transfusion and 4 on hospital day 1 after plasma had been given. After bleeding improved, unfractionated heparin 2 U/kg/h was infused for 24 h as an individualised DIC intervention. No thrombosis or recurrent bleeding was documented. Because evidence for anticoagulation in neonatal DIC is limited, this intervention should not be interpreted as routine treatment or as evidence of efficacy (6, 8, 9).

Intravenous immunoglobulin (total dose, 2.5 g) on days 2–4 and methylprednisolone 4.8 mg every 12 h on days 9–13 were given empirically while immune-mediated thrombocytopenia was considered. Maternal platelet count was normal and the infant's platelet-associated antibody test was negative, but specialised maternal platelet-antibody and maternal, paternal, and infant human-platelet-antigen testing was not performed. The immediate platelet increase followed platelet transfusion, whereas counts fluctuated during and after immunoglobulin and subsequently rose during the period that included corticosteroid treatment; their independent effects could not be determined (10). Ursodeoxycholic acid, ademetionine, and bifendate were used as supportive treatment for cholestatic liver dysfunction. Dobutamine 5 μg/kg/min for 3 days provided short-term haemodynamic support for intermittent bradycardia, relatively low admission blood pressure, and structural shunt-related cardiac findings. Lactate decreased from 2.4 to 1.3 mmol/L, heart rate returned to the normal range, and positive-pressure support was discontinued during combined treatment.

### 2.4 Follow-up and outcomes

At discharge on day 21, platelet count was 88 × 10^9^/L, haemoglobin 111 g/L, body weight 3.35 kg, spontaneous breathing was stable, and hepatosplenomegaly persisted. The skin had a dark greenish discolouration, milk intake was 60 mL every 3 h, and neurological examination showed no obvious abnormality. Because the family requested discharge before the planned assessment was completed, discharge length, head circumference, and same-day hepatobiliary and coagulation results were unavailable. The parents requested discharge because of concerns about prognosis and medical expenses. After the biochemical and molecular reports became available, they were counselled that the findings supported probable neonatal-onset Gaucher disease but did not establish the diagnosis or inheritance pattern. Enzyme replacement therapy and its limited effect on primary neurological disease were discussed but treatment was not initiated. No in-person follow-up occurred. Telephone follow-up documented death at 6 months; the intervening course, terminal records, and certified cause of death were unavailable. The death could not be attributed to Gaucher disease or used to infer a specific subtype from the available evidence. The clinical chronology is summarised in Table 1.

**Table 1:** Timeline of the episode of care, diagnostic investigations, interventions, and outcome.

Phase	Time point	Key findings and management
Prenatal	11–16 weeks' gestation	Maternal rubella immunoglobulin M and G reported positive on three occasions; no avidity, seroconversion, or independent confirmatory testing.
Presentation	Birth to 13 h	Term male, 3.35 kg; petechiae, coffee-ground/bloody gastric drainage, hepatosplenomegaly; platelets 45 × 10^9^/L and fibrinogen 0.67 g/L before transfer.
Early admission	Days 1–6	Platelets 25 × 10^9^/L; infant rubella immunoglobulin G positive/immunoglobulin M negative; CPAP, meropenem/ampicillin, platelet transfusion, plasma already given, and 24-h low-dose heparin. Microbiological investigations were negative; antibiotics stopped on day 6.
Immune-directed treatment	Days 2–13	Intravenous immunoglobulin on days 2–4 and methylprednisolone on days 9–13 for possible immune-mediated thrombocytopenia; independent treatment effects could not be determined.
Progressive disease	Days 13–19	Persistent cytopenias, cholestasis, liver injury, hyperferritinaemia, hepatosplenomegaly, and fluctuating coagulopathy; four HLH-2004 criteria documented at different time points.
Gaucher-directed tests	Day 14	Glucocerebrosidase 0.96 μmol/L/h (reference interval 1.19–22.23); lyso-Gb1 963.66 ng/mL (reference <17.41); *GBA1* sample collected. No independent repeat or cellular enzyme assay.
Discharge	Day 21	Platelets 88 × 10^9^/L, haemoglobin 111 g/L, weight 3.35 kg; stable breathing with persistent hepatosplenomegaly. Parents requested discharge.
Molecular report	After discharge	*GBA1* c.1265_1319del reported as apparently homozygous and pathogenic.
Segregation	After discharge	Variant detected heterozygously in the father and not in the mother; biallelic involvement, inheritance, and recurrence risk unresolved.
Outcome	Age 6 months	Death reported by parents; intervening course, terminal records, and certified cause unavailable.

## 3 Discussion

### 3.1 Gaucher-directed evidence and diagnostic certainty

The evidence had different levels of certainty. Hepatosplenomegaly, cytopenias, cholestasis, and liver injury were compatible with neonatal-onset Gaucher disease but were not specific, and the absence of definite Gaucher cells in bone marrow did not exclude the disorder. The apparent mismatch between the modestly reduced dried-blood-spot glucocerebrosidase activity (0.96 μmol/L/h, approximately 19% below the laboratory lower reference limit) and the more than 55-fold elevation of lyso-Gb1 requires cautious interpretation. Importantly, this degree of reduction relative to the DBS reference limit is not equivalent to 19% residual enzyme activity and cannot be compared directly with diagnostic thresholds established for leukocyte or fibroblast homogenates. The two measurements also assess different biological dimensions: the DBS assay measures *ex vivo* hydrolysis of an artificial substrate in a small whole-blood specimen at a single time point, whereas lyso-Gb1 is an accumulated downstream metabolite reflecting systemic disturbance of glucosylceramide catabolism. Their magnitudes therefore need not be proportional, and residual *in vitro* activity does not reliably predict disease severity (11).

DBS glucocerebrosidase activity may be influenced by applied blood volume, haematocrit, cellular composition, drying, storage, extraction, assay conditions, and recent transfusion. Although the laboratory reported acceptable sample quality, these factors cannot be reconstructed retrospectively, and the single result was not repeated or confirmed in leukocytes or fibroblasts. Conversely, the markedly elevated lyso-Gb1 provided strong evidence that glucosylceramide metabolism was disturbed, but it did not identify the underlying mechanism or independently establish biallelic GBA1 disease (11, 12). A biological discordance may occur when enzyme activity measured against an artificial substrate does not reflect lysosomal function *in vivo*. Saposin C deficiency is particularly relevant because impaired presentation of the natural substrate can produce a Gaucher-like phenotype and marked lyso-Gb1 elevation despite normal or only mildly reduced measured glucocerebrosidase activity (2, 11, 13). Alternatively, an undetected second GBA1 variant, copy-number change, or GBA1/GBAP1-related rearrangement could account for true Gaucher disease despite a misleading DBS result. Analytical error and other lysosomal disorders associated with smaller lyso-Gb1 elevations cannot be excluded from a single measurement; however, the magnitude observed here would be difficult to explain by simple GBA1 heterozygosity alone. Thus, the discordance strengthens suspicion of a glucosylceramide-storage pathway disorder but does not confirm its molecular cause. Resolution would require repeat lyso-Gb1 measurement, leukocyte or fibroblast glucocerebrosidase assay, comprehensive GBA1 structural analysis, and PSAP testing.

### 3.2 Differential diagnosis

Congenital rubella remained relevant because of the prenatal serology and overlapping phenotype, but maternal immunoglobulin M positivity in an asymptomatic pregnant woman may be false positive or persistent, maternal immunoglobulin G does not date infection, and neonatal immunoglobulin G may be maternally derived. Negative neonatal immunoglobulin M and blood metagenomic sequencing did not exclude congenital rubella; the latter was not a validated rubella-specific confirmatory test. Confirmation would have required appropriately timed rubella reverse-transcription polymerase chain reaction from infant respiratory or urine specimens and age-appropriate longitudinal serology. Because these tests were not performed, neither rubella nor a possible coexisting condition could be assigned confidently to the cardiac, hearing-screening, ocular, or cutaneous findings (4, 14). The hearing screen result was not followed by diagnostic testing, and the recorded ocular findings were not characteristic of rubella retinopathy.

The most relevant alternative lysosomal disorders were saposin C deficiency, acid sphingomyelinase deficiency, Niemann–Pick disease type C, and infantile lysosomal acid lipase deficiency (2, 13, 15–18). Saposin C deficiency was particularly important because it can produce Gaucher-like substrate accumulation and marked lyso-Gb1 elevation despite preserved glucocerebrosidase activity *in vitro*, while biallelic GBA1 involvement and a cellular enzyme defect were not established in this infant. Acid sphingomyelinase deficiency can present with neonatal hepatosplenomegaly, cytopenias, and liver dysfunction. Neonatal cholestasis and hepatosplenomegaly can precede neurological manifestations of Niemann–Pick disease type C. Absence of adrenal calcification reduced but did not eliminate concordance with infantile lysosomal acid lipase deficiency. GM1 gangliosidosis, galactosialidosis, mucolipidosis II, mucopolysaccharidosis VII, free sialic acid storage disease, and combined prosaposin deficiency were less concordant but not excluded (15, 19, 20). No targeted lysosomal enzyme or biomarker panel, urine oligosaccharide or glycosaminoglycan testing, or broad genetic panel with copy-number analysis was performed. Thus, routine amino-acid, acylcarnitine, organic-acid, and ammonia screening did not resolve the lysosomal differential diagnosis.

### 3.3 Genetic interpretation

The apparently homozygous GBA1 deletion and discordant parental result did not prove two pathogenic alleles. A second-allele deletion with apparent hemizygosity, allele dropout, a GBA1/GBAP1-related rearrangement or analytical interference, uniparental isodisomy, parental mosaicism, or a *de novo* event remained hypotheses rather than established mechanisms (21, 22). The available report did not permit distinction among true homozygosity, hemizygosity, compound allelic involvement, or an analytical artefact. Orthogonal copy-number analysis would be needed to identify a deletion or duplication not resolved by the original method; single-nucleotide-polymorphism array or family-based informative-marker testing could assess isodisomy and biological relationships; and trio sequencing or long-read analysis could help resolve GBA1/GBAP1-related architecture and phase. Because none of these studies was performed, the allelic configuration, inheritance mechanism, and possibility of a *de novo* or mosaic event remained undetermined.

### 3.4 Phenotype, treatment, and limitations

The case is best described as probable Gaucher disease with a neonatal presentation and undetermined subtype. Hydrops, ichthyosis, a collodion membrane, bulbar dysfunction, seizures, abnormal tone, and neurological deterioration were not documented during the short admission, but their absence did not exclude severe disease (2, 3, 23). During hospitalisation the phenotype was predominantly hepatosplenic, haematological, and cholestatic, rather than the hydrops, skin, respiratory-failure, or early neurological pattern described in some perinatal-lethal cases. Longitudinal neurological and developmental data were unavailable, preventing distinction among acute neuronopathic, perinatal-lethal, and other neonatal phenotypes. The later death could not be attributed to Gaucher disease or used to assign a subtype. Enzyme replacement therapy can improve visceral and haematological manifestations of confirmed Gaucher disease but does not cross the blood–brain barrier (1, 2, 24–26); its potential effect in this unconfirmed case cannot be inferred.

A strength of the report is the chronological integration of infectious, haematological, inflammatory, biochemical, and genetic findings from real-world neonatal practice. The transition from suspected congenital infection to a probable lysosomal disorder demonstrates why each evidentiary layer should be graded separately. Limitations were incomplete rubella testing; single-occasion dried-blood-spot enzyme and biomarker results; unresolved GBA1 segregation; incomplete HLH, immune-thrombocytopenia, hearing, ophthalmic, and alternative lysosomal evaluation; and absence of post-discharge records or a certified cause of death. Measurements were obtained at different time points and after transfusion, antimicrobial, immunoglobulin, corticosteroid, anticoagulant, and supportive interventions, so they should not be reconstructed as a single untreated diagnostic snapshot. The empirical therapies were sequential or concurrent and should not be interpreted as evidence of disease-specific efficacy. The principal lesson is to preserve diagnostic uncertainty when multisystem neonatal disorders produce overlapping findings and incomplete confirmatory evidence.

## 4 Patient perspective

After the biochemical and molecular reports became available, the parents were informed that the combined findings supported probable neonatal-onset Gaucher disease but that definitive confirmation, neurological prognosis, inheritance, and recurrence risk remained uncertain. They were advised that enzyme replacement therapy may improve visceral and haematological manifestations but would not treat primary neurological disease. Concerns about prognosis and medical expenses contributed to their decision to decline further inpatient treatment and request discharge.

## 5 Conclusion

Markedly elevated lyso-Gb1, a compatible neonatal phenotype, modestly reduced dried-blood-spot enzyme activity, and a pathogenic GBA1 variant report supported probable neonatal-onset Gaucher disease. However, independent biochemical confirmation and unequivocal biallelic GBA1 involvement were absent, and other neonatal lysosomal disorders were not systematically excluded. The subtype, allelic configuration, inheritance mechanism, recurrence risk, and cause of death remained undetermined. This case therefore contributes primarily as an illustration of diagnostic reasoning under uncertainty: a compatible phenotype, a highly suggestive biomarker, a modest single enzyme abnormality, and discordant segregation should be integrated but should not be collapsed into a definitive diagnosis. In neonates with overlapping infectious, haematological, inflammatory, hepatic, and metabolic findings, explicitly documenting what was established, what was only supportive, and what was not tested is essential for clinical communication and genetic counselling.

## Data Availability

The original contributions presented in the study are included in the article. Additional raw clinical records are not publicly available because of patient privacy.
